# MiR-1906 Attenuates Neuropathic Pain in Rats by Regulating the TLR4/mTOR/ Akt Signaling Pathway

**DOI:** 10.1515/tnsci-2019-0031

**Published:** 2019-08-07

**Authors:** Xianhai Fang, Huacheng Zhou, Shaopeng Huang, Jinfeng Liu

**Affiliations:** 1 Department of pain clinic, The Second Affiliated Hospital of Harbin Medical University, Harbin, China.150086; 2 Department of pain clinic, The Fourth Affiliated Hospital of Harbin Medical University, Harbin, China150081

**Keywords:** Apoptosis, Chronic constriction injury, Inflammatory mediators, miR-1906, Neuropathic pain

## Abstract

**Background:**

This study determined the role of miR-1906 in neuropathic pain and proliferation in neuronal cells using a chronic constriction injury (CCI)-induced neuropathic pain (NP) rat model.

**Methodology:**

NP was induced by CCI. Animals were divided into a sham group, an NP group, and a miR-1906 mimic group, which received 500 nmol/kg of a miR-1906 mimic intrathecally for 10 consecutive days following surgery. The effect of miR-1906 agomir was determined by estimating the thermal and mechanical withdrawal latency; an enzyme-linked immunosorbent assay (ELISA) was used to determine the concentration of proinflammatory mediators. Western blotting and reverse-transcription polymerase chain reaction (RT-PCR) were used to determine protein expression in the spinal tissues of the CCI-induced neuropathic pain rat model.

**Results:**

Administration of miR-1906 agomir increased the mechanical and thermal withdrawal latency period and the levels of inflammatory mediators compared with the NP group. Western blotting showed that treatment with miR-1906 agomir attenuated the levels of Akt, mTOR, TLR-4, and PI3K proteins in the spinal tissues of the CCI-induced neuropathic pain model. TLR-4 and NF-κB gene expression was lower in the miR-1906 agomir group than in the NP group.

**Conclusion:**

miR-1906 gene stimulation reduced neuropathic pain by enhancing Akt/nTOR/PI3K and TLR-4/NF-κB pathway regulation.

## Introduction

Neuropathic pain (NP) causes several clinical symptoms, including hyperalgesia, spontaneous pain, and allodynia [1]. The clinical application of conventional therapies for NP management reduces pain levels by only 60% [2]. The pathogenesis of NP involves changes to the cell cycle, and its inhibition in spinal cord injury has been shown to decrease the occurrence of neuropathic pain [3]. Several pathways are involved in the control of the cell cycle, including the PI3K/Akt/mTOR pathway [4]. In this process, the mTOR and Akt proteins are activated by the formation of triphosphoinositide from diphosphoinositide by PI3K [5], the mTOR protein controls cell proliferation, and Akt regulates cell survival [6]. The involvement of inflammatory cytokines in the pathogenesis of NP has been well documented; the concentration of proinflammatory mediators is enhanced under the conditions of NP and neuroinflammation [7]. TLR-4 plays a role in the development of NP by stimulating cytokine production and stimulating NF-κB expression, linking immunity and inflammation [8].

MicroRNAs (miRs) are small, non-coding RNAs that have recently been discovered to play a major role as regulators of the development and progression of neuronal damage [9]. Several clinical and preclinical studies have suggested that miR expression is altered in injured neuronal tissues due to changes in the blood flow [10]. miR expression is considered to be a marker of acute ischemic stroke [11]; a recent study used miR-1906 to attenuate cerebral stroke [12]. However, the role of miR-1906 in the development of neuroinflammation and NP remains unclear. Therefore, in this study, we explored the role of miR-1906 in NP.

## Material and Methods

### Animals

Male Sprague–Dawley rats (weight, 200–230 g) were purchased from the Experimental Animal Center at Tongji Medical College, China. Standard guidelines (humidity, 60 ± 5%; temperature, 24 ± 3°C) were followed, and the animals were maintained at a 12-hr light/dark cycle. All protocols for this study were approved by the Institutional Animal Care and Use Committee of the Second Affiliated Hospital of Harbin Medical University, China.

### Experimental Procedure

All animals were anaesthetised with a 60-mg/kg dose of pentobarbital for intrathecal catheter implantation. The catheter was inserted at the level of the spinal cord lumbar enlargement in the subarachnoid space, following the method of [Please insert name and add reference number.]. The animals were separated for a recovery period of 3 days.

NP was induced by chronic constriction injury (CCI)m as described in a previous study [13]. Briefly, all animals were anaesthetised and an incision was made in the left limb to expose the sciatic nerve. Chromic gut sutures (4-0) were used to ligate the nerve in three places and silk sutures were used to stitch the muscle. Infection was prevented by the administration of antibiotics.

The miR-1906 mimic used in this study was obtained from Sigma Aldrich (St. Louis, MO, USA; catalogue no. MLMIR0883). The miR-1906 mimic was delivered using the *MaxSuppressor In Vivo RNA*-LANCEr II RNA delivery system (Bioo

Scientific Co., Austin, TX, USA) according to the manufacturer’s protocol. Animals were divided into three groups: sham, NP, and miR-1906 mimic. The miR-1906 mimic group received 500 nmol/kg of the mimic intrathecally for 10 consecutive days following surgery.

### Determination of mechanical and thermal hyperalgesia

The Hargreaves method was used to determine thermal hyperalgesia [14]. In this procedure, radiant heat (intensity, 20 U) is focused on the mid-plantar area of the hind paw by placing it on a plexiglass cubicle. The time between heat activation on the hind paw and paw withdrawal is then estimated. A pinprick test was also used to determine mechanical hyperalgesia following the method described by [Please insert name and reference number.]. Briefly, a bent gauge needle was applied to the paw with sufficient intensity to induce a withdrawal response, and the time to paw withdrawal was estimated.

### Estimation of inflammatory cytokines

Spinal cord tissue sample homogenates were used to determine the levels of interleukin (IL)-1β, IL-6, and tumour necrosis factor (TNF)-α. Enzyme-linked immunosorbent assay (ELISA) kits were used to estimate the levels of inflammatory cytokines according to the manufacturer’s instructions (Sangon Biotech Co. Ltd., Shanghai, China).

### Western blot assay

Protein samples were isolated from homogenised tissues and quantified using a bicinchoninic acid (BCA) assay kit (Sigma Aldrich). We used 10% sodium dodecyl sulphate–polyacrylamide gel electrophoresis (SDS–PAGE) to separate the proteins. Proteins were then transferred to a nitrocellulose membrane using an electroblotting technique. The membrane was blocked using a 5% blocking solution (non-fat milk) and then incubated in a blocking buffer with the following primary antibodies overnight at 4°C: anti-TLR-4, Akt, PI3K, anti-mTOR, and anti-β actin. The following day, goat secondary antibody conjugated with horseradish peroxidase (HRP) was added to the blocking buffer (1:1,000, non-fat milk), and a chemiluminescence kit (Thermo Fisher Scientific, Shanghai, China) was used to detect the proteins.

### Reverse-transcription polymerase chain reaction (RT-PCR)

RNA was isolated from separated spinal cord tissue using TRIzol Reagent (Thermo Fisher). The RevertAid First Strand cDNA Synthesis Kit (Thermo Fisher) was used to reverse-transcribe the RNA. Specific primers (Table 1) were mixed with RT-2 SYBR green master mix to evaluate gene expression by RT-PCR. The procedure used for all samples was as follows: 98°C for 2 min, followed by 25–40 cycles of 98°C for 10 s, then 55°C for 5 s, and 72°C for 20 s. mRNA expression levels were calculated according to relative standard curves, which were generated by plotting the quantification cycle (Cq) against the log amount of total cDNA added to the reaction. Relative target gene expression levels were determined using the 2^–ΔΔCq^ method.

**Table 1:** Primers used in this study.

Primer		Sequence
mTLR-4	Forward	TAGACACTGTCGCCATGCCT
	Reverse	CGCTGGTGCCTTCGCTATGGT
NF-κB	Forward	5^′^GCAGATGGCCCATACCTTCA-3^′^
	Reverse	5^′^-CACCATGTCCTTGGGTCCAG-3^′^
mGAPDH	Forward	GGCATATGTGAAGCAGACGC
	Reverse	GGAACACTGCTGGTAGGAGAG

### Statistical analyses

All data are expressed as means ± standard error of the mean (SEM) (N = 10). We performed one-way analysis of variance (ANOVA) using the GraphPad Prism ver. 6.1 software (GraphPad Software Inc., San Diego, CA, USA). Following a significant result, means were compared using Dunnett’s *post hoc* test. Statistical significance was set at a level of P < 0.05.

## Results

### miR-1906 agomir ameliorated hyperalgesia

**Figure 1:**
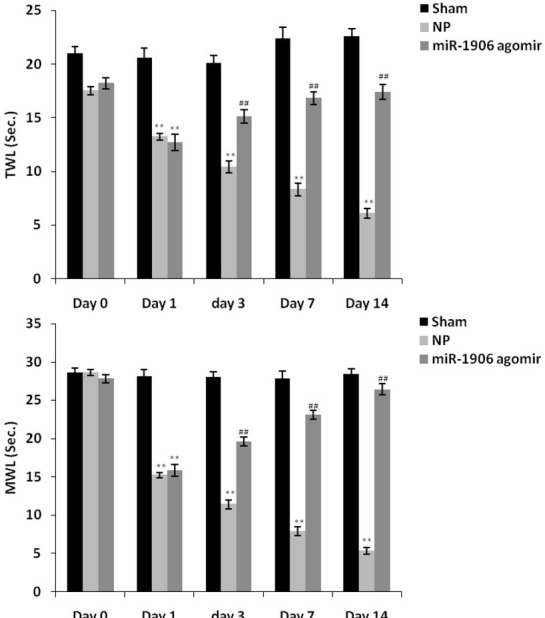
miR-1906 agomir attenuated thermal and mechanical withdrawal latency in a chronic constriction injury (CCI)-induced neuropathic pain (NP) rat model. Data are means ± standard error of the mean (SEM) (N = 10). ^**^P < 0.01 compared with the sham group; ^##^P < 0.01 compared with the NP group.

Rats in the NP group exhibited decreased mechanical and thermal withdrawal latency compared to those in the sham group (Fig. 1). However, administration of miR-1906 agomir enhanced mechanical and thermal withdrawal latency compared to the NP group.

### miR-1906 agomir ameliorated proinflammatory mediator levels

**Figure 2:**
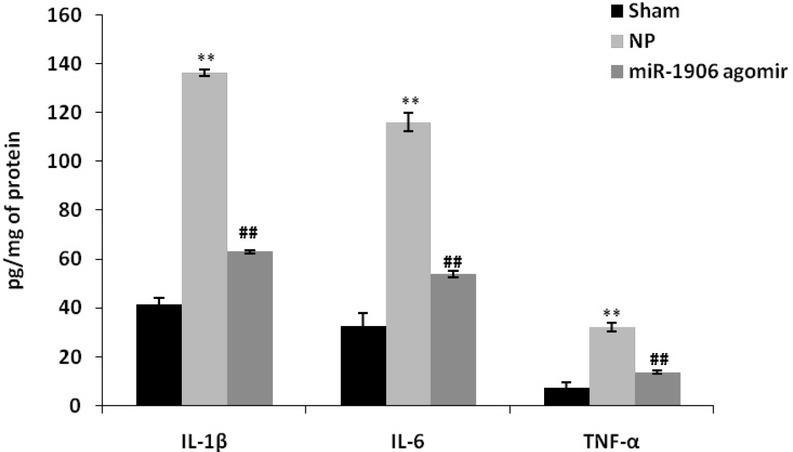
miR-1906 agomir attenuated spinal tissue concentrations of inflammatory cytokines in the CCI-induced NP rat model. Data are means ± SEM (N = 10). ^**^P < 0.01 compared with the sham group; ^##^P < 0.01 compared with the NP group.

The levels of proinflammatory mediators, including IL-1β, IL-6, and TNF-α, were estimated the spinal tissues of the CCI-induced NP rat model (Fig. 2). Spinal tissue concentrations of IL-1β, IL-6, and TNF-α were higher in the NP group than in the sham group, and they were lower in the miR-1906 agomir group than in the NP group.

### miR-1906 agomir ameliorated the expression of TLR-4, Akt, PI3K, and mTOR

**Figure 3:**
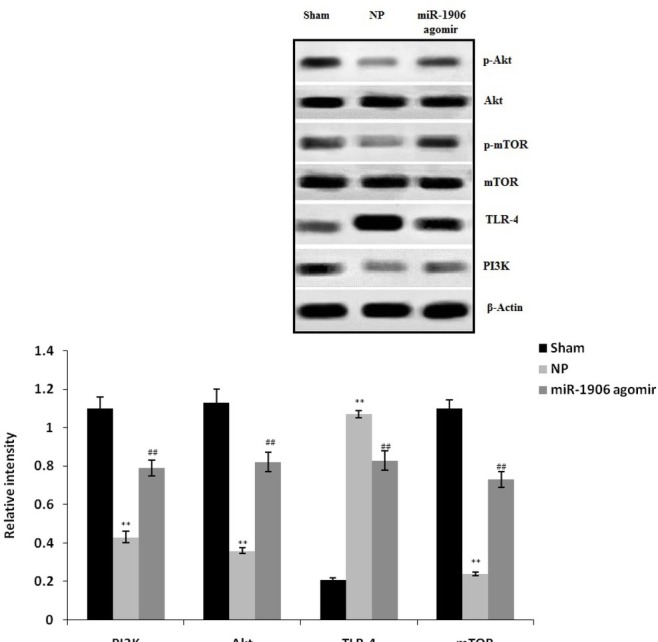
Western blotting results show that miR-1906 agomir attenuated the expression of TLR-4, Akt, PI3K, and mTOR in the spinal tissues of the CCI-induced NP rat model. Data are means ± SEM (N = 10). ^**^P < 0.01 compared with the sham group; ^##^P < 0.01 compared with the NP group.

The expression levels of TLR-4, Akt, PI3K, and mTOR were estimated in the spinal tissues of the CCI-induced NP rat model by Western blotting (Fig. 3). The expression levels of Akt, PI3K, and mTOR proteins were enhanced and that of TLR-4 protein was reduced in the NP group compared with the sham group. However, treatment with miR-1906 agomir attenuated the expression of TLR-4, Akt, PI3K, and mTOR in spinal tissues.

### miR-1906 agomir ameliorated mRNA gene expression of NF-κB and TLR-4

**Figure 4:**
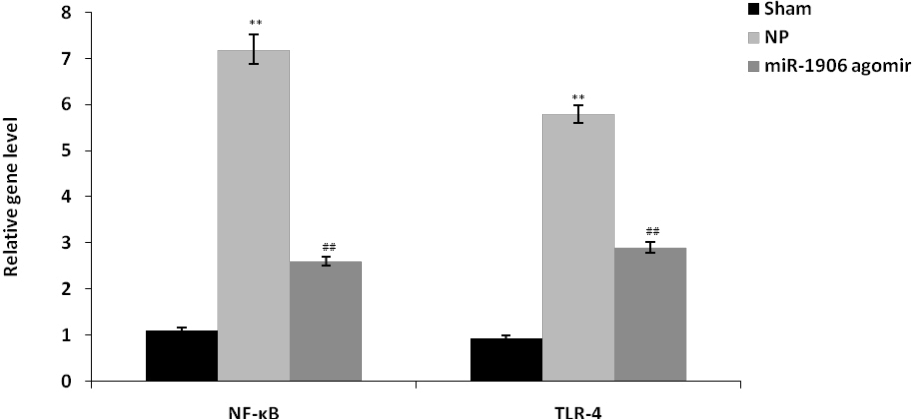
miR-1906 agomir attenuated mRNA gene expression of NF-κB and TLR-4 in spinal tissues of the CCI-induced NP rat model. Data are means ± SEM (N = 10). ^**^P < 0.01 compared with the sham group; ^##^P < 0.01 compared with the NP group.

The mRNA expression levels of the NF-κB and TLR-4 genes in spinal tissues of the CCI-induced NP rat model are shown in Fig. 4. Spinal tissue NF-κB and TLR-4 expression levels were higher in the NP group than in the sham group. However, treatment with miR-1906 agomir significantly decreased NF-κB and TLR-4 expression compared with the NP group (P < 0.01).

## Discussion

NP is a common health problem caused by dysfunction of the somatosensory nervous system. However, the management of NP with conventional drugs remains challenging. Therefore, in this study, we explored the role of miR-1906 in NP regulation by estimating the thermal and mechanical withdrawal latency and the concentrations of proinflammatory mediators following miR-1906 treatment in a CCI-induced NP rat model. Western blotting and RT-PCR were used to determine protein expression in the spinal tissues of the rat model.

It has been well documented that the sensitivity of nociceptive terminals can be stimulated by inflammation mediators [15]. Several cytokines play a role in the pathogenesis of NP, including TNF-α and interlukines 1 β; these have been reported in a previous CCl animal model [16]. Drugs used in the management of NP reduce hyperalgesia responses by reducing interlukine levels [17]. Antagonists of the IL-1 receptor have shown sufficient potency to manage hyperalgesia. The miR-1906 gene has been documented to reduce the levels of inflammatory mediators [18]. The results of our study confirm that administration of miR-1906 agomir to activate the miR-1906 gene reduces hyperalgesia by attenuating inflammatory cytokine levels.

These cytokines stimulate the TLR-4 protein, which has been shown to play a role in pain development in several neuronal injury models and also in inducing NP. The results of several previous studies have revealed that TLR-4 enhances NF-κB levels, thereby inducing NP [19]. The results of the current study show that miR-1906 treatment ameliorates NF-κB and TLR-4 protein levels to reduce NP, which is consistent with the findings of previous studies [20]. Spinal cord injury can induce several proteins that contribute to the development of injury, causing NP [21]. The Akt, mTOR, and PI3K proteins regulate cell proliferation [4]; our results show that the stimulation of the miR-1906 gene by treatment with miR-1906 agomir enhanced the expression of these proteins, thereby offering a new therapeutic candidate for the treatment of NP following spinal cord injury.

## Conclusion

The results of our study demonstrate that miR-1906 agomir reduced NP by ameliorating the levels of inflammatory mediators in a CCI-induced NP rat model. Administration of the miR-1906 gene enhanced the proliferation of neuronal cells by regulating the Akt/nTOR/PI3K and TLR-4/NF-κB pathways.
